# Improving the Performance of a Graphite Foil/Polyaniline Electrode Material by a Thin PEDOT:PSS Layer for Application in Flexible, High Power Supercapacitors

**DOI:** 10.3390/ma13245791

**Published:** 2020-12-18

**Authors:** Zuzanna Zarach, Konrad Trzciński, Marcin Łapiński, Anna Lisowska-Oleksiak, Mariusz Szkoda

**Affiliations:** 1 Faculty of Chemistry, Department of Chemistry and Technology of Functional Materials, Gdańsk University of Technology, Narutowicza 11/12, 80-233 Gdańsk, Poland; trzcinskikonrad@gmail.com (K.T.); alo@pg.edu.pl (A.L.-O.); 2 Faculty of Applied Physics and Mathematics, Gdańsk University of Technology, Narutowicza 11/12, 80-233 Gdańsk, Poland; marcin.lapinski@pg.edu.pl

**Keywords:** conducting polymers, electrodeposition, supercapacitors, conjugated polymers composites, polyaniline stability

## Abstract

In this study, we present a novel strategy for enhancing polyaniline stability and thus obtaining an electrode material with practical application in supercapacitors. A promising (graphite foil/polyaniline/poly(3,4-ethylenedioxythiophene):poly(styrenesulfonate) GF/PANI/PEDOT:PSS) electrode material was characterized and used in the construction of a symmetric supercapacitor that provides an outstanding high power density. For this purpose, the electropolymerization of PANI was carried out on a graphite foil and then a thin protective layer of PEDOT:PSS was deposited. The presence of the nanometer PEDOT:PSS layer made it possible to widen the electroactivity potential range of the electrode material. Moreover, the synergy between materials positively affected the amount of accumulated charge, and thus the thin PEDOT:PSS layer contributed to enhancing the specific capacity of the electrode material. The electrochemical performance of the GF/PANI/PEDOT:PSS electrode, as well as the symmetrical supercapacitor, was investigated by cyclic voltammetry and galvanostatic charge/discharge cycles in 1 M H_2_SO_4_ at room temperature. The fabricated electrode material shows a high specific capacitance (*C*_sp_) of 557.4 Fg^−1^ and areal capacitance (*C*_areal_) of 2600 mF·cm^−2^ in 1 M H_2_SO_4_ at a current density of 200 mA·cm^−2^ (~4 A·g^−1^). The supercapacitor performance was studied and the results show that a thin PEDOT:PSS layer enables cycling stability improvement of the device from 54% to 67% after 10,000 cycles, and provides a high specific capacity (159.8 F·g^−1^) and a maximum specific power (18,043 W·kg^−1^) for practical applications.

## 1. Introduction

In recent years, researchers have been motivated to develop sustainable and renewable energy devices due to the exponential utilization of fossil fuels and subsequently related environmental issues. Moreover, the emergence of new technologies and materials has gained considerable interest in electrochemical energy storage devices such as supercapacitors and batteries. Since the first patent in 1957 by the General Electric Company [1], the academic research and industrial production of supercapacitors have been intensified. Since then, supercapacitors potential applications have been extended to a range of areas including power backup systems, electric vehicles, digital communication devices and storing the energy generated by solar cells [2,3,4,5]. Progress in supercapacitor design is considered as one of the most significant and innovative ways towards reliable electrical energy storage. This is due to their high power density, long cycle life, low maintenance cost, and environmental safety, as well as secure operating conditions.

In general, there are two major varieties of supercapacitors, namely, electrochemical double-layer capacitors (EDLCs) and pseudo-capacitors, depending on the charge storage mechanism. In EDLCs energy is stored at the electrode/electrolyte interface via the separation of opposite charges, building an inner layer and a diffuse layer at the electrode/electrolyte interface [6]. The stored charge is of electrostatic nature, thus EDLC devices have no kinetic limitations [7] and as for electrode manufacturing in EDLD, mostly carbonaceous materials are exploited [8]. On the contrary, in pseudocapacitors reversible and rapid faradaic reactions, strictly limited to the electrode surface, take place. As the charge is related to faradaic processes, not to electrostatic ones, some kinetic limitations may occur. The nature of pseudocapacity is linked with active surface groups, taking part in electron charge transfer. The term pseudocapacity describes the behavior of electrode materials which have the electrochemical signature of a capacitive electrode [7,9], that is, the straightforward relation of the charge stored with the potential window range [10]. Generally, pseudocapacitors can deliver significantly more specific capacitance and energy density than EDLC [11,12]. Electrode materials have a major impact on the electrochemical performance of supercapacitors. In general, they can be divided into carbon-based materials, transition metal oxides and conducting polymers [8,13].

As for pseudo-capacitive electrode materials, metal oxides, conducting polymers (CPs), and recently g-C3N4 are widely used [14,15,16,17,18,19]. In particular, conducting polymers have become a major subject of electrochemical research because of their unique and advantageous properties, thus enabling their possible application in various fields. Among them, polyaniline (PANI) is one of the most promising CP due to its emerging features, such as easy synthesis, doping and de-doping simplicity, chemical stability, as well as good environmental stability, mechanical versatility, and low cost [20,21]. The major disadvantage of PANI working as a pseudocapacitive electrode is primarily the lack of stability during the cycles [22]. Moreover, to overcome the electronic conductivity problem, some substantial work was carried out to improve the electronic conduction of PANI electrodes by metal doping [23,24]. However, the use of metals significantly increases technological and material costs and contributes to difficulties in disposing the used capacitors.

Furthermore, in order to obtain conductive polymers with appropriate properties, mixing, composite and copolymerization approaches were fitted. More recently, copolymerization has generated considerable interest in order to produce new conductive copolymers with desired properties that would enable overcoming the drawbacks and shortcomings of the corresponding monomers. Finally, the synthesized copolymer could be presented together with both homopolymer positive features [25]. The thickness and morphology of the materials can be better controlled by preparing homopolymers and copolymers using electrochemical rather than chemical synthesis [26,27]. Some research on the properties of PEDOT and PANI based copolymers has been reported [28,29,30]. In the case of layer-on-layer type polymer composites, synergy between the components can also be expected.

Hence, this paper outlines an approach of supercapacitor fabrication involving carrying out electropolymerization of PANI on graphite foil and eventually depositing a thin layer of PEDOT:PSS on top. Electrochemical deposition of PANI was performed by potentiostatic polymerization in an aqueous solution containing hydrochloric acid and aniline. Afterwards, the electrode was covered by a thin layer of PEDOT:PSS from an aqueous solution containing PSS anions as a source of counterions, once again using potentiostatic electrodeposition. The electrode material was characterized using SEM, Raman, and X-ray photoelectron spectroscopy (XPS) at each stage of electrodeposition. Characterization of electrochemical properties of the electrode material, as well as the symmetric supercapacitor, was made by performing cyclic voltammetry and galvanostatic charge-discharge tests. The aim of this research was to make a valuable contribution to the enhancement of the stability of polyaniline, which was obtained through the deposition of a thin layer of PEDOT:PSS, and the development of a supercapacitor of high specific power. The synergy between the polymers improved the performance of the electrode material and thus the overall performance of the symmetric supercapacitor, characterized not only by a high specific power, but also a great specific capacity and capacity retention.

## 2. Materials and Methods

### 2.1. Chemicals

Aniline (p.a. Chempur, Karlsruhe, Germany), sodiumpolystyrene sulfonate (Sigma Aldrich, Saint Louis, MO, USA) and 3,4-ethylenedioxythiophene (99%, Acros Organics, Geel, Belgium) were used as received. Sulfuric acid (p.a. 95%) and hydrochloric acid (p.a. 35%), used for electrolyte preparation, were purchased from POCH. Graphite foil (thickness 0.4 mm, 99.8%, Alfa Aesar, Haverhill, MA, USA) was used as the conductive substrate.

### 2.2. Fabrication of the GF/PANI/PEDOT Electrode Material

**Figure 1:**
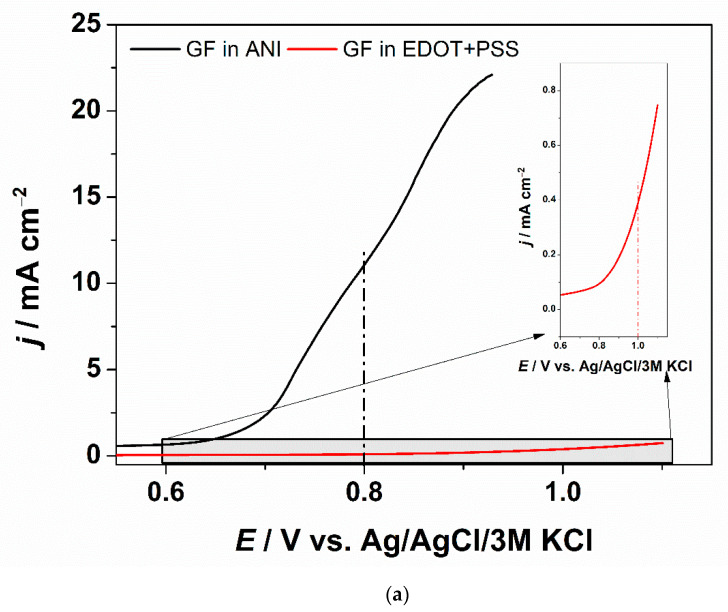

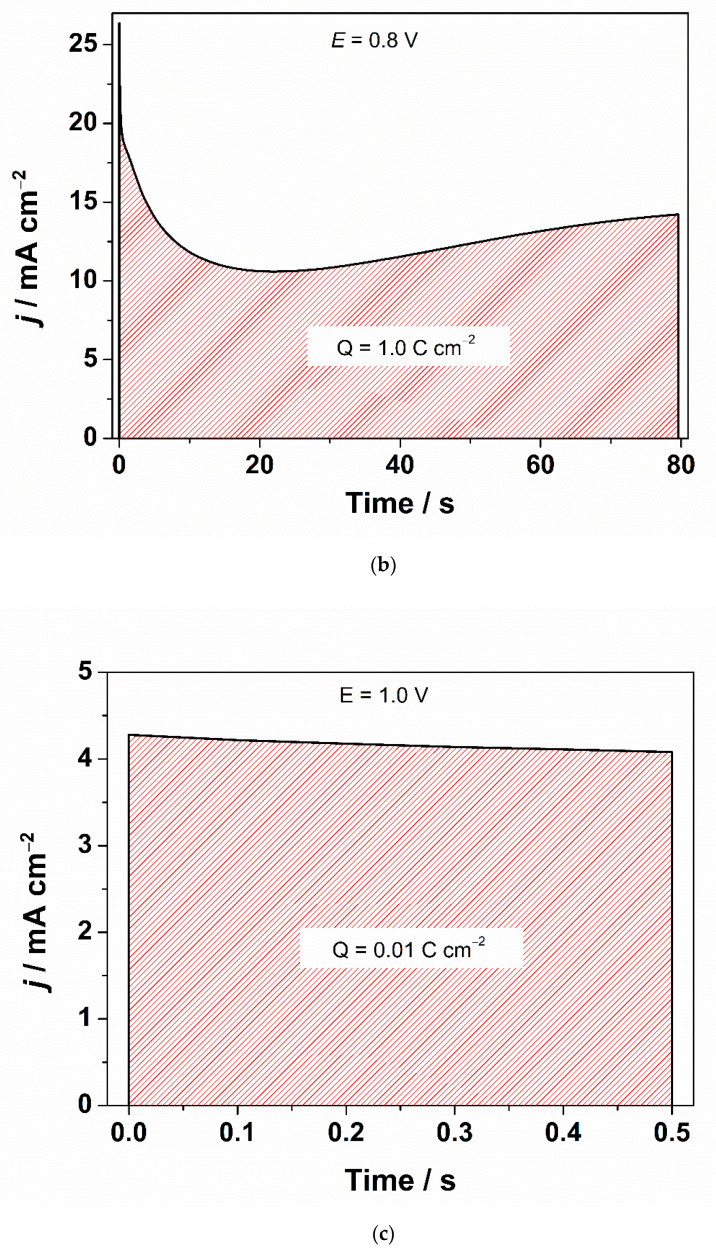
(**a**) Linear sweep voltammetry (LSV) curves with marked potential value for electrodeposition of PANI and PEDOT:PSS; inset: zoomed LSV curve for PEDOT:PSS; Chronoamperometry curves recorded during (**b**) PANI (at *E* = 0.8 V) and (**c**) PEDOT:PSS (at *E* = 1.0 V) electropolymerization on GF and GF/PANI, respectively.

Before starting the deposition process, graphite foil was subjected to a purification process by placing the samples in isopropanol for 10 min. After a series of optimization experiments the electrochemical deposition of PANI was performed in an aqueous solution containing 3 M HCl and 1 M ANI, by potentiostatic polymerization of 0.85 V vs. Ag/AgCl/3 M KCl consuming a charge of 1.5 C·cm^−2^. Next, the obtained GF/PANI sample was covered by a thin PEDOT:PSS layer via electrodeposition (*E* = 1.05 V vs. Ag/AgCl/3 M KCl consuming a charge of 0.01 C·cm^−2^) from 0.015 M EDOT with 0.1 M PSSNa electrolyte. The electrodeposition potential was chosen on the basis of linear sweep voltammetry curves, which are presented in Figure 1a, and were recorded during anodic polarization of the GF electrode in electrolytes containing monomers, aniline, and EDOT, respectively.

The mass loading of the polymers was measured by the weight difference of the electrode material (dried at room temperature) before and after electropolymerization, using an Analytical Balance RADWAG XA 82/220.4Y PLUS (Radom, Poland) with an accuracy of 0.01 mg. The results of optimization experiments are presented in Supplementary Figures S1–S4. The main goal was to establish the charge flow during electrodeposition of PANI and PEDOT:PSS that would enable obtaining of highest values of the parameters, including specific capacity and capacity retention. As a result, polyaniline was electrodeposited on graphite foil with a 1.5 C·cm^−2^ charge, whereas PEDOT:PSS was electrodeposited on GF/PANI with a 0.01 C·cm^−2^ charge.

A symmetric supercapacitor was constructed by combining two GF/PANI/PEDOT:PSS electrodes and placing a fiberglass separator soaked in 1 M H_2_SO_4_ aqueous electrolyte between them. In the next step, the casing foil was welded on three sides using a plastic foil welder, and finally the setup was sealed using a vacuum packing machine (CAS CVP-350/MS, Hertogenbosch, The Netherlands).

### 2.3. Physicochemical Characterization Techniques

The morphology of the electrode materials was studied by a scanning electron microscope (Quanta 3D FEG, Fei Company, Hillsboro, OR, USA). The X-Ray Photoelectron Spectroscopy (XPS) analysis was carried out using a 128-channel, hemispherical Argus spectrometer (Omicron Nanotechnology, Limburg an der Lahn, Germany). The spectroscope was equipped with a Mg Kα source, operated at 15 kV and 300 W. The pass energy was 20 eV and the spot size diameter was ca. 2 mm × 2 mm. The instrument was calibrated on metallic gold. The obtained spectra were analyzed using CasaXPS 2.3.18 software on a Shirley background. The micro-Raman spectrometer Renishaw InVia (Wotton-under-Edge, UK) with excitation of samples by means of an argon laser emitting at the wavelength of 514 nm was used for Raman spectra collection.

### 2.4. Electrochemical Studies

Electrochemical measurements of the electrode materials and pure graphite foil were performed by cyclic voltammetry (CV) and galvanostatic charge-discharge tests (GCD) using a potentiostat–galvanostat (BioLogic VSP 2078, Seyssinet-Pariset, France). Electrochemical impedance spectroscopy measurements were performed in the frequency range between 20 kHz and 1 Hz in 1 molar sulfuric acid, using FRA software (Version 4.9). The electrode materials were studied in a three-electrode electrochemical cell. The Ag/AgCl/3.0 M KCl electrodes and Pt mesh served as the reference and the counter electrode, respectively. Different electrodes, i.e., GF, GF/PEDOT:PSS, GF/PANI and GF/PANI/PEDOT:PSS were used as the working electrode. The electrochemical measurements were carried out in contact with a 1 M H_2_SO_4_ aqueous solution. The charge/discharge measurements were carried out with a current density in a range from 1 to 8 A·g^−1^ in a polarization range: −0.2 to 0.9 V. For all measurements, the electrolyte was initially purged with argon for 30 min in order to remove oxygen and the experiments were carried out under argon atmosphere. Electrochemical measurements for the symmetric supercapacitor, i.e., cyclic voltammetry and galvanostatic charge and discharge tests (1000 and 10,000 cycles) were conducted using techniques mentioned above. Cyclic voltammetry was performed at a scan rate of 50 mV·s^−1^ to determine the working voltage window, which was finally fixed at 0.6 V. Charge and discharge measurements were made applying current density values in the range from 1 to 30 A·g^−1^ in the electrochemical voltage range of 0.6 V.

## 3. Results and Discussion

### 3.1. Electrodeposition of Polyaniline and Poly(3,4-ethylenedioxythiophene)

The LSV curves are presented in Figure 1a. As can be seen, both monomers can be oxidized directly on graphite foil within the electrochemical window of water. The chronoamperometry curves recorded during PANI and PEDOT electropolymerization on GF and GF/PANI are presented in Figure 1b,c, respectively.

Electrochemical deposition provides greater deposition control rather than chemical deposition and ensures that PANI nucleation and growth do not occur in solution, but on the surface of the electrode [31]. PANI nucleation in solution is undesirable because this can affect the final product uniformity. Early research [32,33] of the kinetic growth of polyaniline deposited on the electrode under potentiostatic conditions revealed that polyaniline deposition on an electrode surface can be presented by a model that consists of two stages: initial nucleation and growth. Further work has shown greater complexity of the initial nucleation stage. Similarly to the case of polypyrrole [34], the initial step to deposit and to form polyaniline nucleation sites on the electrode surface was not dependent on the potential applied, suggesting that nucleation is possibly not an electrochemical stage during polymerization [35] and that the polymer probably aggregates briefly on the electrode, building oligomers being later precipitated onto the electrode [36], which may be also described as the starting point of the growth process. Nevertheless, it is believed that precipitation occurs during the whole polymerization process, not only at the initial stage [34]. This stage—the growth, may be described by two separate stages. Firstly, the formation of a strongly bound compact layer can be observed and then, the growth of a loosely bound open structure takes place. The kinetics of this process is very likely to be represented by the nucleation model and 2D growth since the lateral rate of growth is much more rapid than the normal one. This effect may be clarified given that, relative to the oxidation of polymer chains, the aniline oxidation on the electrode surface is favored. In comparison, 1D growth of polymer chains and continuous polymer branching demonstrate the growth kinetics of a less compact polymer array. In general, conjugated polymers, when electrodeposited, are known to grow open. This means that their density changes with material thickness and outer parts of the film are less compact in comparison with inner parts, when deposited on a dense solid support [37].

### 3.2. Morphology and Chemical Structure

**Figure 2:**
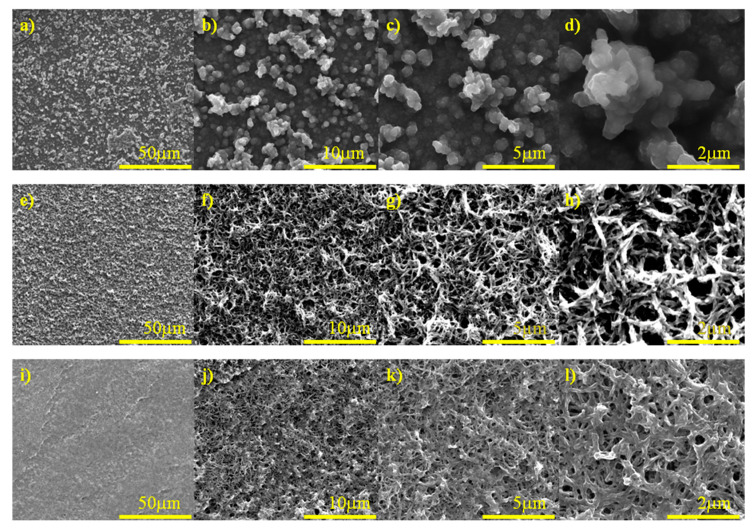
SEM images of (**a**–**d**) GF/PEDOT:PSS, (**e**–**h**) GF/PANI and (**i**–**l**) GF/PANI/PEDOT:PSS.

The morphology of the graphite foil (GF), GF/PEDOT:PSS, GF/PANI and GF/PANI/PEDOT:PSS were investigated by SEM. Figure S5 (in Supplementary Information) shows the top view of a GF for comparison. It is seen that the surface of the GF is quite smooth and small graphite flakes are tightly packed on top. SEM micrographs of the GF electrode after PEDOT:PSS electrodeposition are presented in Figure 2a–d. PEDOT:PSS grown over a graphite foil was in the form of stuck lumps and at the highest magnification we can see that the obtained material had a cauliflower-like structure (Figure 2a–d). On the contrary, polyaniline formed a porous structure (Figure 2e–h), which uniformly covered the surface of the graphite foil. The combination of both materials can be seen in Figure 2i–l—a reduction in porosity can be observed as a result of PEDOT:PSS lumps joining the PANI structure. Additionally, cross section SEM images of GF/PANI/PEDOT:PSS and GF/PANI electrodes are presented in Supplementary Figure S6. After comparing the thickness of the deposited polymer layers, which in both cases is practically identical, it can be concluded that a separate PEDOT:PSS layer on the GF/PANI cannot be identified and measured. On the other hand, the SEM images in Figure 2i–l indicate changes in the porosity of the surface. It should be assumed that the PEDOT:PSS polymerization took place in the available space of the PANI’s pores.

**Figure 3:**
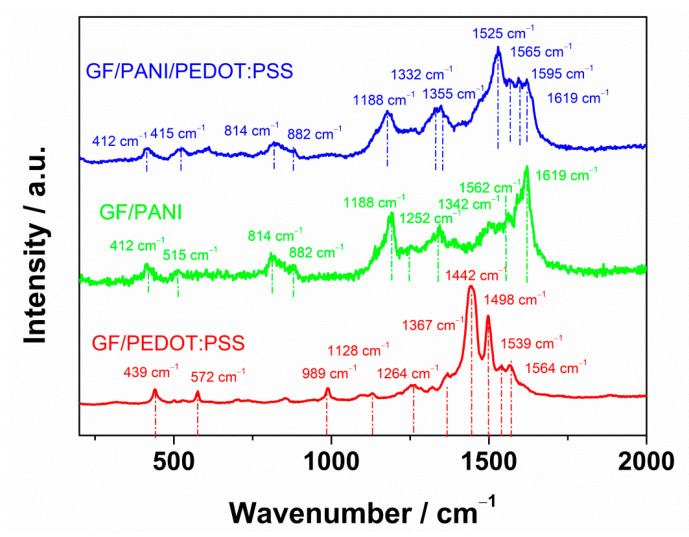
Raman spectra of GF/PEDOT:PSS, GF/PANI and GF/PANI/PEDOT:PSS.

In the Raman spectrum of GF/PEDOT:PSS, shown in Figure 3, typical bands of the polymer were identified. The PEDOT:PSS spectrum exhibits the most intense and well-shaped peak located at 1442 cm^−1^ and also one at 1498 cm^−1^, which refer to symmetrical C_α_=C_β_ vibrations in the thiophene ring. The bands located in the range from 1539 to 1564 cm^−1^ can be assigned to asymmetrical C_α_=C_β_ stretching modes in polymer chains. Furthermore, bands at 1367, 1264 and 1128 cm^−1^ can be found and ascribed to single bonded carbon atoms: C_α_–C_α_’ inner ring stretching, C–O–C ring deformation and C_β_–C_β’_ stretching, respectively [38]. Moreover, some bands can be observed in the region between 989 and 439 cm^−1^, which are due to oxyethylene ring deformations. It is noteworthy that spectra obtained for PEDOT:PSS films typically contain all these features [39,40,41].

The Raman spectrum of GF/PANI is also shown in Figure 3. The most intense and clear band registered at 1619 cm^−1^ reflects the C–C stretching vibration of the benzene ring. Another one, located at 1562 cm^−1^, indicates N–H bending vibrations [42,43]. The stretching vibrations of an intermediate bond C–N^+^ of a polaronic structure occur in Raman spectra with a characteristic frequency around 1342 cm^−1^. Worth mentioning is the fact that researchers do not agree what exact value is the most precise [43,44,45]. Furthermore, the C–N stretching mode of a polaronic unit can be represented by the band at 1252 cm^−1^. The second most intensive band at 1188 cm^−1^ could be assigned to C–H in the plain deformation mode of the protonated emeraldine form of PANI [46,47,48,49]. Finally, some other bands in the region of lower wavenumbers should be pointed out: the peak at 882 cm^−1^ is connected with C–N–C wagging, whereas the band at 814 cm^−1^ describes benzene ring deformations. Moreover, out of plane ring deformations are represented by bands at 515 and 412 cm^−1^ [50,51].

Analyzing the obtained Raman spectra and comparing them with the spectrum obtained for GF/PANI/PEDOT:PSS, it can be concluded that in this case a composite consisting of both polymers was attained. This is due to the fact that, in the spectrum, characteristic bands for each of the polymers can be observed, and some of them are slightly shifted after the superimposition of the signals due to interactions between polymers.

**Figure 4:**
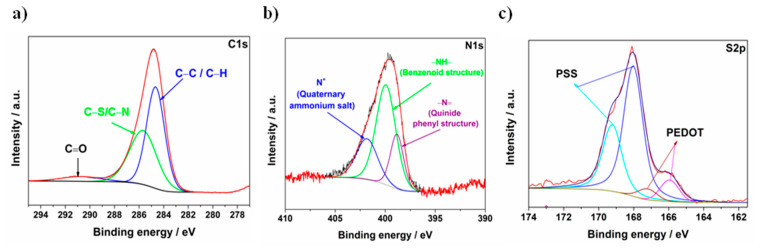
XPS spectra of GF/PANI/PEDOT:PSS: (**a**) C1s, (**b**) N1s and (**c**) S2p.

To determine the composition of the as-prepared GF/PANI/PEDOT:PSS electrode material, XPS was used. The high resolution XPS spectra of C1s, N1s and S2p regions are shown in Figure 4a–c. The XPS spectra of the C1s region can be well fitted with three peaks due to C–C or C–H (284.6 eV), C–N (285.7 eV), and C=O (290.9 eV) [52,53,54]. The N1s peak is observed at about 400 eV, which is quite asymmetric and needs to be deconvoluted. After the deconvolution, N1s shows three peaks, namely, the quinoid phenyl structure (–N=), the benzenoid structure (–NH–) and the quaternary ammonium structure (N^+^) at 398.8, 400.0 and 401.8 eV, respectively, as shown in Figure 4b [52]. The presence of peaks characteristic for nitrogen atoms indicates that the PEDOT:PSS does not form a tight layer on the polyaniline film. Typical S2p core-level spectra of the PEDOT:PSS films are shown in Figure 4c. The XPS spectrum exhibits a high intensity band between 168.0 and 169 eV, which corresponds to the spin-split components of sulfur atoms of the PSS chains. The peak is highly asymmetric, however, as it was reported previously [55], this effect is related to the neutralization of SO_3_^−^ groups by e.g., Na^+^ and H^+^. Moreover, between 166 and 167 eV, two other peaks can be observed, assigned to the sulfur atoms of the PEDOT structure [53,56]. The asymmetry of this peak is probably due to the distribution of differentially charged sulfur atoms in PEDOT. According to the XPS measurements, the PSS:PEDOT ratio can be estimated to be about 4:1. The PSS:PEDOT ratio depends on the method of the film deposition [57]. The scatter of the results presented in the literature is large, even in the case of the same preparation method (electropolymerization). Very thin PEDOT:PSS films are characterized by an excess of PSS—PSS:PEDOT ratio equals to 4.1 [58], while in the case of the thicker layer, the ratio was equal to 0.4 [59].

### 3.3. Electrochemical Characterization

#### 3.3.1. Three-Electrode Configuration

**Figure 5:**
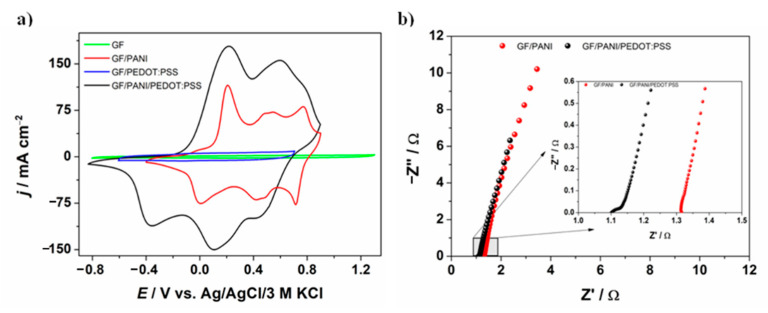
(**a**) Cyclic voltammetry curves (*v* = 50 mV s^−1^) and (**b**) electrochemical impedance spectroscopy curves recorded in a range of frequency between 20 kHz and 1 Hz for different electrode materials in 1 M H_2_SO_4_.

The electrochemical performance of GF/PANI and GF/PANI/PEDOT:PSS electrode materials in a three-electrode system was investigated, using obtained materials as the working electrode. The comparison of the CV curves of a GF/PANI and a GF/PANI/PEDOT:PSS composite is presented in Figure 5a. The CV curves for GF and GF/PEDOT:PSS are also presented for comparison. Both electrodes (GF/PANI and a GF/PANI/PEDOT:PSS) exhibited characteristic peaks of redox couples of polyaniline [60]. The current density of a GF/PANI/PEDOT:PSS electrode is larger than that of the GF/PANI electrode. Comparing the current densities of polyaniline and PEDOT:PSS separately, it can be concluded that it is not simply the sum of the current utilized for oxidation/reduction of both polymers. The synergy between materials should positively affect the amount of the accumulated charge. It is worth mentioning that PEDOT:PSS plays an important role in increasing electroactivity of the material, as well as it may contribute to expanding the specific surface area of the electrode. Moreover, the presence of PEDOT:PSS affects the widening of the electroactivity potential range as PEDOT:PSS is electroactive in a more cathodic potential range and its pseudocapacitive activity takes place in the negative potential region up to −0.9 V. On the other hand, PANI pseudocapacitive activity mostly covers the positive potential region. Moreover, the PEDOT:PSS film is known to behave as a material permeable for cations in the potential range of the PEDOT doped state [61,62]. Electrochemical quartz microbalance studies proved the selectivity of ionic transport as limited to hydrated cations [61]. Thus, the PEDOT:PSS cover on top of polyaniline can act as an ion-selective membrane permeable for protons.

In the Nyquist plots, presented in Figure 5b, electrochemical impedance spectroscopy measurements, which were performed at a rest potential of the electrode, are presented. The slope of the curves indicates the capacitive nature of the electrodes, while the value of the intersection of the *x*-axis indicates the value of the internal resistance of the electrolyte. Moreover, the reactance (the imaginary part of impedance) can be represented by the Equation (1):

(1) $$Z^{\prime \prime }\;=\;-\frac{1}{2\pi fC}$$

Thus, it can be concluded that the highest capacitance values are obtained for the GF/PANI/PEDOT:PSS electrode, which is consistent with cyclic voltammetry results.

**Figure 6:**
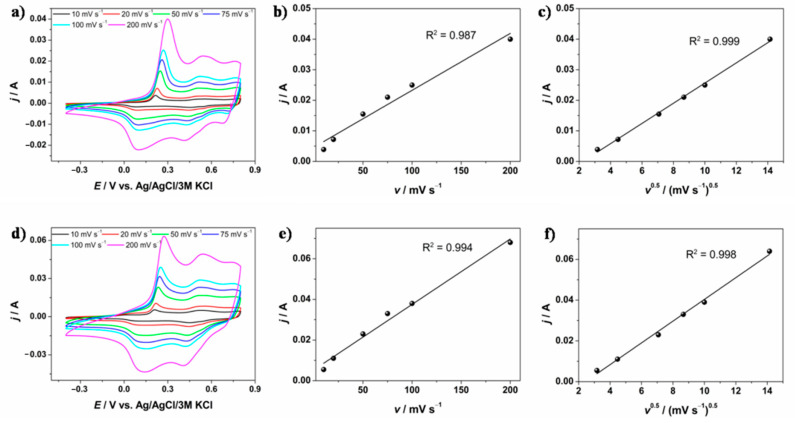
Cyclic voltammetry curves registered at different scan rates for (**a**) GF/PANI and (**d**) GF/PANI/PEDOT:PSS; plots of *j* = *f(v*) and *j* = *f*(*v*^1/2^) at *E* = 0.3 V for GF/PANI (**b**,**c**) and GF/PANI/PEDOT:PSS (**e**,**f**).

To investigate what type of storage charge mechanism determines the energy storage, cyclic voltammetry was performed at different scan rates for two electrode materials: GF/PANI and GF/PANI/PEDOT:PSS. The results are presented in Figure 6a,d, respectively. Analyzing the shape of the obtained curves, as well as the redox couple activity, one may predict that pseudocapacitance has a significant contribution in the energy storage process. A better insight into the charge transfer process was obtained by plotting the curves *j* = *f*(*v*) (Figure 6b,e) and *j* = *f*(*v*^1/2^) (Figure 6c,f), using the current values at a defined potential value (*E* = 0.3 V) for both electrode materials. For a GF/PANI electrode, better linear dependence was obtained for *j* = *f*(*v*^1/2^) fitting, which is consistent with the much greater contribution of pseudocapacitance (diffusion controlled charge transfer). On the contrary, fitting results for a GF/PANI/PEDOT:PSS showed that both pseudocapacitance and electrostatic adsorption on the surface take place and have an impact on the overall capacity of the electrode material. PEDOT:PSS is widely regarded as a material that stores energy using pseudocapacitive mechanisms. However, experimental evidence and theoretical model results presented by Volkov et al. [63] indicate that PEDOT:PSS uses the electrical double layer mechanism. This is consistent with the results obtained for the GF/PANI/PEDOT:PSS electrode material, i.e., with an increase in the contribution of the capacitive mechanism of energy storage (Figure 6e). Moreover, as mentioned above, for GF/PANI/PEDOT:PSS, it is not simply the sum of the current utilized for oxidation/reduction of both polymers. The synergy between PANI and PEDOT:PSS may be associated with the accumulation of an additional charge in the vicinity of the PANI-PEDOT:PSS interface and thus a significant increase in total capacity is observed (see Figure 5a). The synergistic effect has an influence on the enhancement of the capacitive mechanism contribution to the energy storage process.

**Figure 7:**
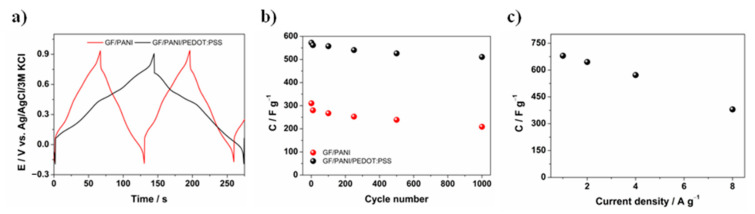
(**a**) Galvanostatic charge/discharge curves recorded for GF/PANI and GF/PANI/PEDOT:PSS electrode materials; (**b**) capacity retention after 1000 cycles of GF/PANI and GF/PANI/PEDOT:PSS electrode materials; (**c**) specific capacity in the function of current density applied.

Galvanostatic charge/discharge measurements, presented in Figure 7a, were performed at a current density of 200 mA·cm^−2^ (~4 A·g^−1^) in a 1 M H_2_SO_4_ aqueous solution. Typical capacitive behavior with almost symmetric charge/discharge curves is observed for both types of electrodes. The specific capacitance (*C*_sp_) was calculated using the galvanostatic charge/discharge curves provided in Figure 7a and Equation (2):

(2) $$C_{\mathrm{sp}}=\;\frac{I\cdot dt}{dV\;\cdot A\;\left(or\;m\right)}$$

where *I* is the applied discharge current, *t* is the discharge time, *V* is the discharge voltage and *A* is the active area in cm^2^ (*m*—mass of the active material). The specific capacitance (after 100 cycles) of the electrodes was calculated as 1.2 F·cm^−2^ (267.4 F·g^−1^, *m* = 0.0046 g) and 2.6 F·cm^−2^ (557.4 F·g^−1^, *m* = 0.0048 g) for the GF/PANI and the GF/PANI/PEDOT:PSS electrode material, respectively. The GF/PANI/PEDOT:PSS electrode exhibits about two times higher specific capacitance than GF/PANI. Thus, the presence of the thin PEDOT:PSS layer improves the stability and widens the electroactivity potential range of the composite into more negative potentials. The specific capacitance of GF/PANI/PEDOT:PSS, with a comparatively high loading of the polymer (4.8 mg·cm^−2^) and measured at a high discharge current density (0.02 A·cm^−2^/~4 A·g^−1^), is also higher than or comparable to the values recently reported for polymer composites (see Table 1).

The cycling measurements were conducted at 200 mA·cm^−2^ (~4 A·g^−1^) in 1 M H_2_SO_4_ for 1000 times for the GF/PANI and GF/PANI/PEDOT:PSS electrodes in order to investigate their prolonged charge/discharge behavior (Figure 7b). The GF/PANI electrode loses 33% of its specific capacitance (from 310.3 to 208.8 F·g^−1^). Capacity retention at the level of 89% specific capacitance (510.8 of 572.2 F·g^−1^) after 1000 cycles was observed for GF/PANI/PEDOT:PSS, indicating that this electrode material is more stable than GF/PANI. Therefore, it can be concluded that the presence of a thin PEDOT:PSS layer not only affects the improvement of capacity, but also has a positive influence on the stability of the composite. For comparison, the multiple charge/discharge cycles, as well as an example of CP curves for pure GF and GF/PEDOT:PSS electrode material, are presented in Supplementary Figure S7. As can be seen, their electrochemical stability is high, but the capacity for these materials is definitely lower compared to GF/PANI and GF/PANI/PEDOT:PSS. On the basis of previous electrochemical quartz microbalance (EQMC) studies, one may conclude that a thin PEDOT:PSS film acts as a permeable membrane, selectively promoting proton transport and entirely blocking SO_4_^2−^ and HSO_4_^−^ ion transport throughout the film, as given in Ref. [61]. The influence of anions on electroactivity of the PANI film was studied using the EQMC method by Song et al. [82]. Authors precisely evidenced that the water molecule, as a hydration shell of counter-ions (anions), introduced into the polymer matrix during electrosorption under the doping process, causes the degradation of PANI. The electrochemical degradation products are usually identified as *p*-quinone/*p*-hydroquinone and *p*-quinone imine/*p*-aminophenol redox couples [83]. Similar to PEDOT:PSS thin films, a carbonaceous cover was once used to enhance electrochemical stability of polyaniline and polypyrrole [84]. In the case of combining polyaniline with PEDOT:PSS, Liu et al. [85] indicated that the enhanced stability of polyaniline was mostly due to –SO_3_^−^H^+^ groups in poly(styrenesulfonate), which serve as an internal reservoir of protons necessary for PANI protonation, particularly providing a high local H^+^ concentration and eventually affecting the overall electrochemical reversibility and activity.

The specific capacitance of the GF/PANI/PEDOT:PSS electrode measured at different discharge current densities, ranging from 1 to 8 A·g^−1^ is presented in Figure 7c. The specific capacitance was 720 and 330 F·g^−1^ at current densities of 1 and 8 A·g^−1^, respectively, and thus it can be concluded that the discharge current density has a significant effect on the electrode capacity, as was previously reported [64,78].

#### 3.3.2. Two-Electrode Configuration

**Figure 8:**
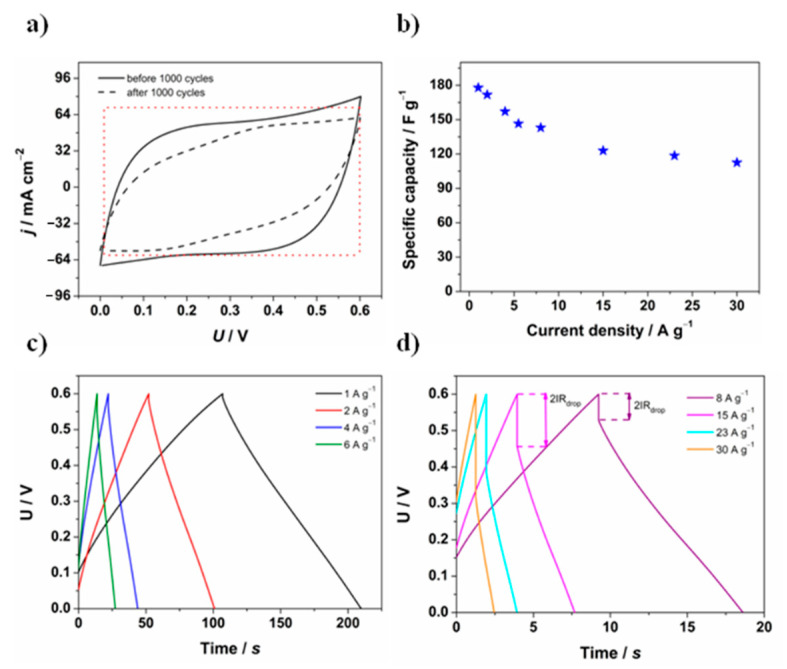
(**a**) Cyclic voltammetry curves (*v* = 50 mV·s^−1^) recorded for a GF/PANI/PEDOT:PSS symmetric supercapacitor before and after 1000 charge and discharge cycles; (**b**) specific capacity as a function of the current density applied in charge/discharge measurements for a GF/PANI/PEDOT:PSS symmetric supercapacitor; (**c**,**d**) charge and discharge curves at a different current density applied for a GF/PANI/PEDOT:PSS symmetric supercapacitor.

In the final stage of the work, a symmetric supercapacitor was fabricated using two GF/PANI/PEDOT:PSS electrodes. The electrochemical performance of the device was investigated using cyclic voltammetry and galvanostatic charge and discharge tests. At first, the cyclic voltammetry study was performed to determine the working voltage window and then charge and discharge measurements were made applying current density values in the range from 1 to 30 A·g^−1^ (Figure 8b). Based on the measurements, the electrochemical voltage range was set at 0.6 V, which is presented in Figure 8a—in the proposed voltage window, the shape of the voltammetry curve most closely corresponds to the curve of an ideal capacitor, i.e., a rectangular curve shape—displayed as a dotted red curve. After 1000 cycles, a slight change in the shape of the curve was observed, however it did not significantly change the overall performance of the device.

Charge/discharge measurements were performed at different current densities and even under high current density values (Figure 8b) they maintained a symmetric quasi-triangular shape, which provided a high specific capacitance, which may be observed in Figure 8c,d. While increasing the current density value, a decrease in specific capacity is detected. This phenomenon is mainly associated with the rise of the internal resistance value and thus the *2IR*_drop_, which relates to the internal resistance of the electrode material [86,87]. Its occurrence is frequently said to be caused by the low electron and ionic transfer rate [88]. Apart from the huge impact on the specific capacity, the higher value of ESR contributes to the reduction in power density of the device and consumption of energy during charging.

**Figure 9:**
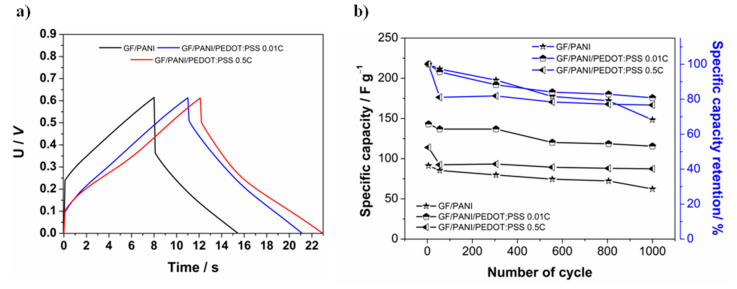
(**a**) Exemplary galvanostatic charge and discharge curves for supercapacitors assembled from different electrode materials; (**b**) Specific capacity and specific capacity retention of different symmetric supercapacitors as a function of the number of cycles performed.

After determining the operating voltage window, galvanostatic charge and discharge tests were performed. For every device constructed, the measurement was conducted with the same current value applied: 0.03 A (4.8 mA·cm^−2^). The obtained results were compared with those acquired from testing symmetric supercapacitors assembled from GF/PANI and GF/PANI/PEDOT:PSS (*Q* = 0.5 C·cm^−2^) electrode materials, presented in Figure 9. The summary of the most important parameters is presented in Table 2.

As can be seen from Figure 9a, a GF/PANI device is characterized by a much greater ohmic drop value, which is strictly coupled with the increase in the internal resistance value and eventually affects the total power density of the supercapacitor. Moreover, the distortion of the curves’ shape from an ideally triangular one indicates the occurrence of pseudocapacitive behavior [89,90]. From Figure 9b it can be concluded that not only the PEDOT:PSS layer improves the specific capacity value, but also significantly contributes to enhancing the stability of polyaniline and thus the overall performance of the device. Comparing the two devices comprising of an electrodeposited layer of PEDOT:PSS, it can be observed that despite a higher charge flow value and thus a bigger amount of the active material, it did not result in increasing the specific capacity value. Moreover, it did not contribute to improvement of the supercapacitor capacity retention. Relatively better results were obtained for the material consisting of the thinner PEDOT:PSS layer, even with the much higher current density applied. This result may have been due to the fact that polyaniline has a major contribution in pseudo-capacity occurrence and thus, increasing the thickness of the PEDOT:PSS layer, eventually blocks the effect caused by polyaniline properties and inhibits anion transfer and promotes the transfer of protons [61,62]. Notwithstanding, there is no doubt that PEDOT:PSS presence hinders the process of degradation of polyaniline by slowing it down.

**Figure 10:**
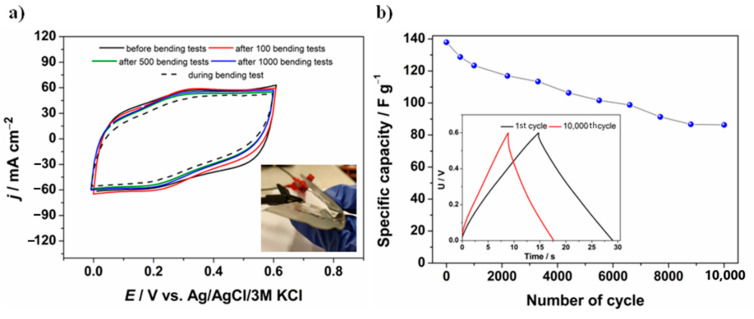
(**a**) Cyclic voltammetry curves recorded for a symmetric GF/PANI/PEDOT:PSS supercapacitor before and after bending tests, as well as during bending (*v* = 50 mV·s^−1^); inset: image of the bent GF/PANI/PEDOT:PSS symmetric supercapacitor; (**b**) Specific capacity as a function of the number of cycles performed for a symmetric GF/PANI/PEDOT:PSS supercapacitor after 10,000 bending tests; inset: charge/discharge curves at 1st and 10,000th charge/discharge cycle.

In order to further explore the advantages of the proposed electrode material and its behavior as an element of the supercapacitor construction, the device has been subjected to multiple bending tests in different directions, as presented in the inset in Figure 10a and then compared with the supercapacitor before bending tests (initial). Cyclic voltammetry data in a potential window between 0 and 0.6 V vs. Ag/AgCl/3 M KCl with the scan rate 50 mV·s^−1^ were plotted in Figure 10a. Moreover, cyclic voltammetry measurements were made after the device had been bent for 1000 times, as well as during the bending procedure (see Video S1). As can be observed, there is no significant difference between individual CV curves, even when the measurement was taken during bending of the device, indicating high flexible properties of the proposed electrode material. The GF/PANI/PEDOT:PSS symmetric supercapacitor displayed excellent mechanical and electrochemical stability under bending conditions, which is an essential feature in practical applications. Moreover, after 1000 bending tests being performed, the performance of the device was investigated using multiple charge/discharge measurements. After 10,000 cycles, the device retained about 63% of the initial capacity (see Figure 10b). As can be seen in the inset of Figure 10b, the symmetrical triangular shape of the charge/discharge curve was preserved and the specific capacity value stabilizes at about 87 F·g^−1^.

**Figure 11:**
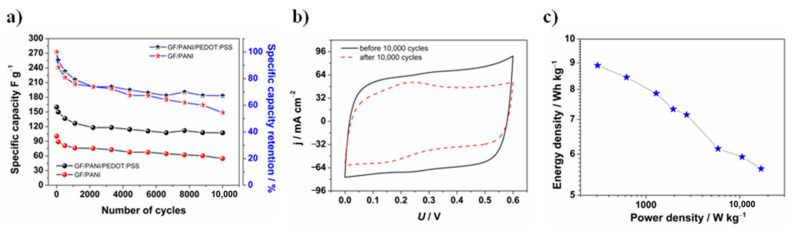
(**a**) Specific capacity retention after 10,000 cycles for a GF/PANI and a GF/PANI/PEDOT:PSS (*Q* = 0.01 C·cm^−2^) symmetric supercapacitor; (**b**) cyclic voltammetry curves (*v* = 50 mV·s^−1^) recorded for a GF/PANI/PEDOT:PSS symmetric supercapacitor before and after 10,000 charge and discharge cycles and (**c**) Ragone plot for a GF/PANI/PEDOT:PSS symmetric supercapacitor (each star indicates the energy density value for calculated power density at different current densities applied).

In the final stage of the work, additional charge and discharge cycles for a GF/PANI/PEDOT:PSS symmetric supercapacitor were performed, presented in Figure 11a, whereas Figure 11b presents cyclic voltammetry curves before and after 10,000 cycles performed. Furthermore, to obtain a comprehensive characteristic, the specific energy and maximum specific power were calculated [87,91,92,93], using Equations (3) and (4), respectively:

(3) $$E_{s}\;=\;\frac{C_{\mathrm{sp}}{\left(U_{\max}\right)}^{2}}{2}\frac{1000}{3600}\;\left[\frac{\mathrm{Wh}}{\mathrm{kg}}\right]$$

(4) $$P_{\max}\;=\;\frac{U_{0}^{2}}{4Rm}\;\left[\frac{W}{\mathrm{kg}}\right]$$

where *C*_sp_ is the specific capacity value, $U_{\max}$ is the voltage range in which discharge takes place; *U*_0_ is the voltage value at the beginning of the discharge (after the ohmic drop), *m* is the mass of the active material, and *R* is the resistance that can be calculated from the ohmic drop according to Equation (5) [92]:

(5) $$R\;=\;\frac{V_{\mathrm{drop}}}{2I}\left[\Omega \right]$$

where *V*_drop_ is the initial ohmic drop of the cell at the beginning of the discharge and *I* is the constant current in the discharging process. It should be noted that apart from the maximum value of specific power, the average-power density could be calculated, using the formula below:

(6) $$P_{s}\;=\;\frac{E_{s}}{\Delta t}\;\left[\frac{W}{\mathrm{kg}}\right]$$

where Δt is the discharge time of the cell.

After 10,000 cycles of charging and discharging of the supercapacitors, the GF/PANI/PEDOT:PSS electrode material enabled achieving of higher specific capacity values than GF/PANI. The most valuable information is that the capacity retention of the device was increased from 54 to 67% due to the presence of a thin PEDOT:PSS layer, which is consistent with the results of measurements performed in a three-electrode system. Moreover, just like after 1000 cycles, the current density remains at high values and it can be seen that the curve did not flatten, maintaining a rectangular-like shape (Figure 11b), confirming the stability of the material and the overall electrochemical performance of the device. At the Ragone plot in Figure 11c, which presents the average-power density and energy density of the device at different current densities, it can be observed that the energy density value is comparable with the reference values (which are presented in Table 3). The average-power density at the highest values of current applied is extremely high, at the same time there is no intense decrease in the energy density of the device. What deserves special mention is that the obtained value of the maximum specific power, determined using Equation (4), is 18,043 W·kg^−1^ at a 5.5 A·g^−1^ current density. It is highly desirable, especially when it comes to power-stabilization applications like backup systems. The overall performance of a GF/PANI/PEDOT:PSS symmetric supercapacitor was compared with properties of the devices described in the literature, which are listed in Table 3.

## 4. Conclusions

A high-performance GF/PANI/PEDOT:PSS flexible electrode material for a supercapacitor was developed by the electrodeposition of PANI and PEDOT:PSS on a graphite foil surface. The supercapacitor electrode exhibits a high capacitance of 2600 mF·cm^−2^ and 557.4 F·g^−1^ at a current density of 200 mA·cm^−2^ (~4 A·g^−1^) in the three-electrode system in 1 M H_2_SO_4_ electrolyte. The high performance mainly corresponds to the creation of synergistic and complementary effects between PANI and PEDOT:PSS while eliminating their individual drawbacks. As for the symmetric GF/PANI/PEDOT:PSS supercapacitor, it exhibits a high specific capacity (159.8 F·g^−1^) and an improvement of cycling stability after 1000 and 10,000 cycles, in comparison with the GF/PANI supercapacitor, which can be observed due to the presence of a thin PEDOT:PSS layer. Moreover, the electrode material and thus the whole device is characterized by high flexibility, which points to the possibility of real applications. The results of the present work could provide new insight into the improvement of the stability of polyaniline and the development of conductive polymer-based flexible supercapacitors with a high specific capacity while ensuring an outstanding capacity retention for practical applications.

## Figures and Tables

**Table 1:** Specific capacitance values comparison of recently reported polymer composites.

Electrode Material	Specific Capacity *C*_sp_	Current Density/Scan Rate	Ref.
SWNT/WO_3_/PANI	28.5 mF·cm^−2^	0.13 mA·cm^−2^	[64]
Polyaniline with Li salt	107 F·g^−1^	1.25 mA·cm^−2^	[65]
PVA/PANI	571 F·g^−1^	5 mV·s^−1^	[22]
Graphene/polyaniline woven	23 mF·cm^−2^	0.1 mA·cm^−2^	[66]
WO_3_/PANI	4.1 mF·cm^−2^	0.02 mA·cm^−2^	[67]
PANI/WO_3_	12 mF·cm^−2^	0.008 mA·cm^−2^	[68]
Hierarchical graphene/polypyrrole nanosheet composites	319 F·g^−1^	2 mV·s^−1^	[69]
Polyaniline nanowires/graphene oxide nanosheets composite	227 F·g^−1^	2 A·g^−1^	[70]
Polyaniline nanotubes	558 F·g^−1^	200 mV·s^−1^	[71]
Graphene/polyaniline composite paper	233 F·g^−1^	2 mV·s^−1^	[72]
Graphene/polyaniline nanofiber composite	210 F·g^−1^	0.3 A·g^−1^	[73]
Metal–Organic Framework/PANI	2.1 mF·cm^−2^	10 mV·s^−1^	[74]
MoS_2_/PANI	552 F·g^−1^	0.5 A·g^−1^	[75]
Graphene@PANI nanoworm composites	488.2 F·g^−1^	0.5 A·g^−1^	[76]
TiO_2_ nanotubes/PANI	740 F·g^−1^	3 A·g^−1^	[77]
cellulose/graphite/polyaniline composite	357 F·g^−1^	80 mA·cm^−2^	[78]
PANI/rGO/CeO_2_	684 F·g^−1^	4 A·g^−1^	[79]
ZnO@MOF@PANI	340.7 F·g^−1^	1.0 A·g^−1^	[80]
PANI/carbon cloth	1800 mF·cm^−2^	1.73 A·g^−1^	[81]
GF/PANI/PEDOT:PSS	2600 mF·cm^−2^ 720 F·g^−1^	200 mA·cm^−2^ 1 A·g^−1^	This work

**Table 2:** Electrochemical performance of the devices assembled from different electrode materials after 1000 charge and discharge cycles.

Electrode Material	Specific Capacity *C*_sp_	Specific Capacity Retention
GF/PANI	91.3 F·g^−1^	68.2%
GF/PANI/PEDOT:PSS 0.01 C·cm^−2^	143.0 F·g^−1^	80.8%
GF/PANI/PEDOT:PSS 0.5 C·cm^−2^	113.9 F·g^−1^	76.6%

**Table 3:** Comparison of the supercapacitors assembled from polyaniline and/or poly(3,4-ethylenedioxythiophene) as electrode material composites.

Electrode Material	Specific Capacity *C*_sp_	Current Density	Energy Density	Power Density	Capacity Retention	Ref.
Ag-PEDOT:PSS/PANI nanofibres	396 F·g^−1^	0.1 mA	86.19 Wh·kg^−1^	-	85.8% after 1000 cycles	[9]
RGO/PEDOT/PANI	302.5 F·g^−1^	1 A·g^−1^	26.89 Wh·kg^−1^	800 W·kg^−1^	99% after 10,000 cycles	[94]
CNFs/PANI	201 F·g^−1^	0.25 A·g^−1^	4.5 Wh·kg^−1^	103 W·kg^−1^	80% after 6000 cycles	[95]
HPC/PANI	69 F·g^−1^	1 A·g^−1^	9.6 Wh·kg^−1^	223 W kg^−1^	78% after 10,000 cycles	[96]
PANI-MWNT	240 F·g^−1^	4 A·g^−1^	12 Wh·kg^−1^	2586.8 W·kg^−1^	93% after 5000 cycles	[97]
GA/RGO/MnO_2_@PANI	122 F·g^−1^	1.5 A·g^−1^	38.12 Wh·kg^−1^	1191 W·kg^−1^	85.8% after 5000 cycles	[98]
rGO/TiO_2_/PEDOT nanocomposite	544 F·g^−1^	50 mA	1.14 Wh·kg^−1^ (at 50 mV/s)	1792 W·kg^−1^ (at 1 000 mV/s)	92.7% after 1000 cycles	[99]
PANI/MWNTs/TiO_2_	270 F·g^−1^	0.4 A·g^−1^	13.5 Wh·kg^−1^ (at 0.4 A·g^−1^)	2034 W·kg^−1^ (at 4 Ag^−1^)	67% after 6000 cycles	[100]
PANI/Mn-TiO_2_nanoparticles	52.5 F·g^−1^	1 A·g^−1^	18.66 Wh·kg^−1^	800 W·kg^−1^	91% after 5000 cycles	[101]
PANI/PEDOT hydrogel	80.8 F·g^−1^	1 mA·cm^−2^	0.63 mWh·cm^−3^	28.42 mW·cm^−3^	83.9% after 5000 cycles	[102]
PEDOT/PANI hydrogel	3.5 F·cm^−3^	1 mA·cm^−2^	0.25 mWh·cm^−3^	107.14 mW·cm^−3^	80.8% after 5000 cycles	[103]
GF/PANI/PEDOT:PSS	159.8 F·g^−1^	5.6 A·g^−1^	7.32 Wh·kg^−1^	18,043 W·kg^−1^	80.8% after 1000 cycles;67% after 10,000 cycles	This work

## Supplementary Materials

The following are available online at https://www.mdpi.com/1996-1944/13/24/5791/s1, Figure S1: Cyclic voltammetry curves (*v* = 50 mV·s^−1^) recorded for GF/PANI/PEDOT:PSS electrode materials in 1 M H_2_SO_4_ with different charges used for electrodeposition of polyaniline (charge during electrodeposition of PEDOT:PSS: 0.01 C·cm^−2^), Figure S2: (**a**) Specific capacity retention after 100 cycles of GF/PANI/PEDOT:PSS electrode materials with different charges used for electrodeposition of polyaniline; (charge used for electrodeposition of PEDOT:PSS: 0.01 C·cm^−2^); (**b**) Specific capacity of a GF/PANI/PEDOT:PSS electrode material as a function of the charge used for electrodeposition of polyaniline (charge used for electrodeposition of PEDOT:PSS: 0.01 C·cm^−2^), Figure S3: Cyclic voltammetry curves (*v* = 50 mV·s^−1^) recorded for GF/PANI/PEDOT:PSS electrode materials in 1 M H_2_SO_4_ with different charges used for electrodeposition of poly(3,4-ethylenedioxythiophene) (charge used for electrodeposition of PANI: 1.5 C·cm^−2^), Figure S4: (**a**) Specific capacity retention after 100 cycles of GF/PANI/PEDOT:PSS electrode materials with different charges used for electrodeposition of poly(3,4-ethylenedioxythiophene) (charge at electrodeposition of PANI: 1.5 C·cm^−2^); (**b**) Specific capacity of a GF/PANI/PEDOT:PSS electrode material as a function of the charge used for electrodeposition of poly(3,4-ethylenedioxythiophene) (charge used for electrodeposition of PANI: 1.5 C·cm^−2^), Figure S5: SEM image of a graphite foil with a (**a**) 1000, (**b**) 5000 and (**c**) 10,000 magnification., Figure S6: Cross section SEM images of (**a**) GF/PANI/PEDOT:PSS and (**b**) GF/PANI electrodes, Figure S7: Exemplary galvanostatic charge/discharge curves of (**a**) GF and (**b**) GF/PEDOT:PSS electrodes; (**c**) Specific capacity vs. cycle number for GF and GF/PEDOT:PSS, Video S1: Performance of bending procedure for a GF/PANI/PEDOT:PSS symmetric supercapacitor.
