# Supplementary material for: Improving the Performance of a Graphite Foil/Polyaniline Electrode Material by a Thin PEDOT:PSS Layer for Application in Flexible, High Power Supercapacitors
Source: Materials (Basel). 2020 Dec 18;13(24):5791. doi: 10.3390/ma13245791 (PMC7766753; doi:10.3390/ma13245791)
Supplement: Supplementary file 1 [file materials-13-05791-s001.zip › materials-1023597-supplementary.docx]

Supplementary Information

Improving the Performance of a Graphite Foil/Polyaniline Electrode Material by a Thin PEDOT:PSS Layer for Application in Flexible, High Power Supercapacitors

Zuzanna Zarach ^1,^*, Konrad Trzciński ^1^, Marcin Łapiński ^2^, Anna Lisowska-Oleksiak ^1^ and Mariusz Szkoda ^1,^*

^1^ Faculty of Chemistry, Department of Chemistry and Technology of Functional Materials, Gdańsk University of Technology, Narutowicza 11/12, 80-233 Gdańsk, Poland; trzcinskikonrad@gmail.com (K.T.); alo@pg.edu.pl (A.L.-O.)

^2^ Faculty of Applied Physics and Mathematics, Gdańsk University of Technology, Narutowicza 11/12, 80-233 Gdańsk, Poland; marcin.lapinski@pg.edu.pl

***** Correspondence: zuzanna.zarach@pg.edu.pl (Z.Z.); mariusz.szkoda@pg.edu.pl (M.S.)

Received: 17 November 2020; Accepted: 15 December 2020; Published: 18 December 2020

At first, the optimization experiments included electrodeposition of polyaniline on graphite foil using different charges of electrodeposition and thus layers of various thickness were obtained (Figure S1). The characteristic peaks derived from redox reactions of polyaniline could be distinguished. Moreover, it can be observed that above 1.5 C cm^−2^ there is no significant increase in a current density when a higher charge is applied.


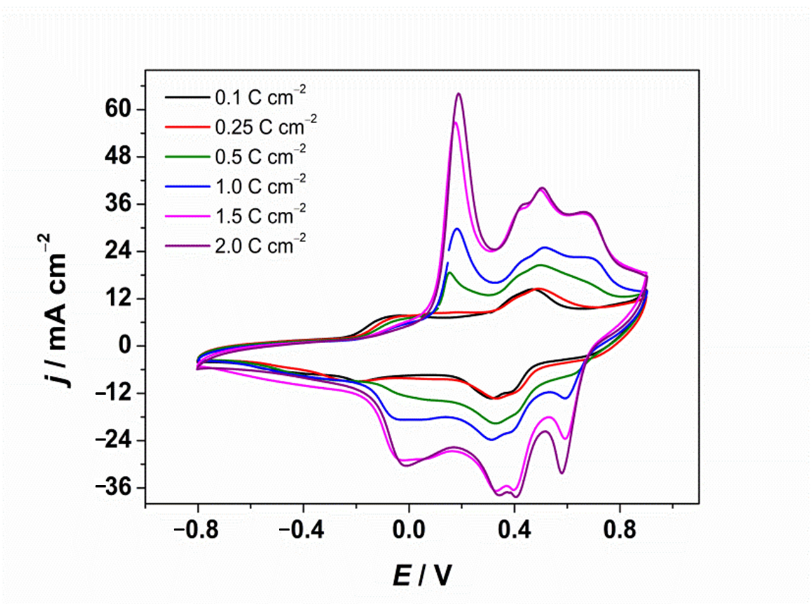


**Figure S1.** Cyclic voltammetry curves (*v* = 50 mV·s^−1^) recorded for GF/PANI/PEDOT:PSS electrode materials in 1 M H_2_SO_4_ with different charges used for electrodeposition of polyaniline (charge during electrodeposition of PEDOT:PSS: 0.01 C·cm^−2^).

For previously prepared GF/PANI/PEDOT:PSS electrodes, galvanostatic charge and discharge tests were conducted in a three electrode configuration. Similarly to the cyclic voltammetry results, the highest values of specific capacity were obtained for higher charge values applied during electropolymerization of PANI (Figure S2a). Moreover, for the initial cycles, a nearly linear relationship between the capacity and the charge can be observed (Figure S2b). For 2.0 C·cm^−2^, after 100 cycles, the specific capacity retention was at 67% (from 390.0 to 261.3 mF·cm^−2^), whereas for 1.5 C·cm^−2^, after 100 cycles, the specific capacity retention was at the level of 75% (from 292.6 to 281.4 mF·cm^−2^). Despite the fact that the value of the specific capacity was lower for 1.5 C·cm^−2^, the stability of the electrode material was better and the material was less degraded. Moreover, the polyaniline layer obtained with the 2.0 C·cm^−2^ deposition charge was so thick that it was being washed out faster and was flowing off the surface. Therefore, the charge flow with 1.5 C·cm^−2^ was chosen.


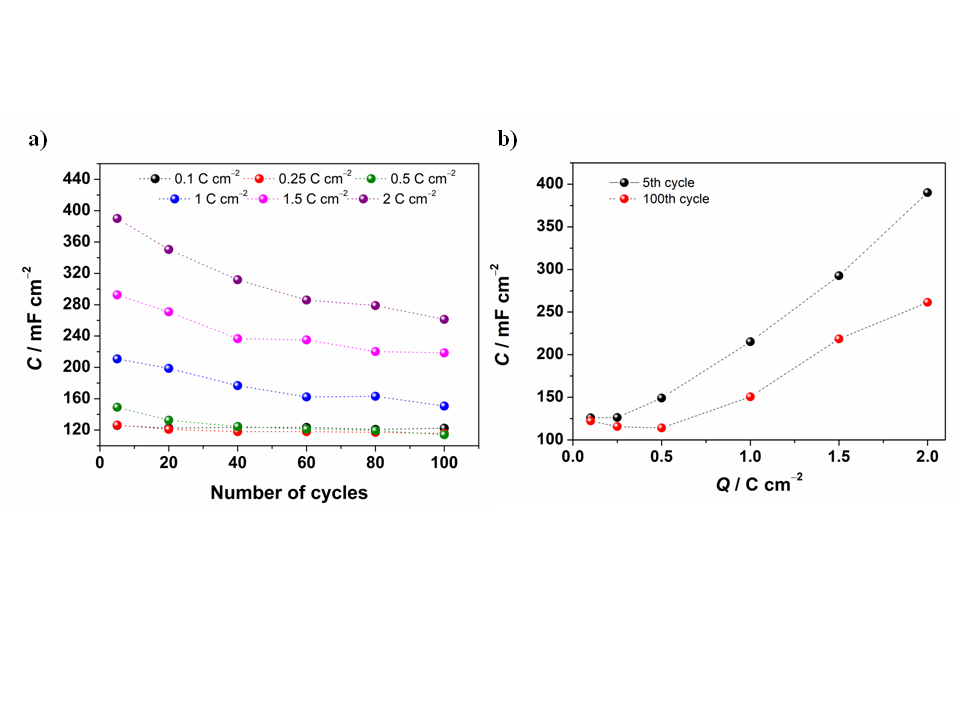


**Figure S2. (a**) Specific capacity retention after 100 cycles of GF/PANI/PEDOT:PSS electrode materials with different charges used for electrodeposition of polyaniline; (charge used for electrodeposition of PEDOT:PSS: 0.01 C·cm^−2^); (**b**) Specific capacity of a GF/PANI/PEDOT:PSS electrode material as a function of the charge used for electrodeposition of polyaniline (charge used for electrodeposition of PEDOT:PSS: 0.01 C·cm^−2^).

The same procedure was used for the determination of the charge used for the electropolymerization of poly(3,4-ethylenedioxythiophene) on a GF/PANI electrode material. As it can be observed at Figure S3, the effect of different charges to the overall capacity is hard to distinguished from voltammetric curves as the current density values are similar for all of the samples. The better insight into the influence of the PEDOT:PSS layer can be gained by looking at the results of galvanostatic charge and discharge tests (Figure S4). It can be concluded that the PEDOT:PSS layer does not play as significant role in increasing a specific capacity value as polyaniline and there is no linear dependency between the charge value and the obtained specific capacity value (Figure S4b)). However, the highest capacity values were obtained for the thinnest PEDOT:PSS layer and the capacity retention after 100 cycles remained at the level of 75%. More intensive investigation over the influence of PANI and PEDOT:PSS on the specific capacity and capacity retention was conducted during two electrode measurements in the subsequent experiments.


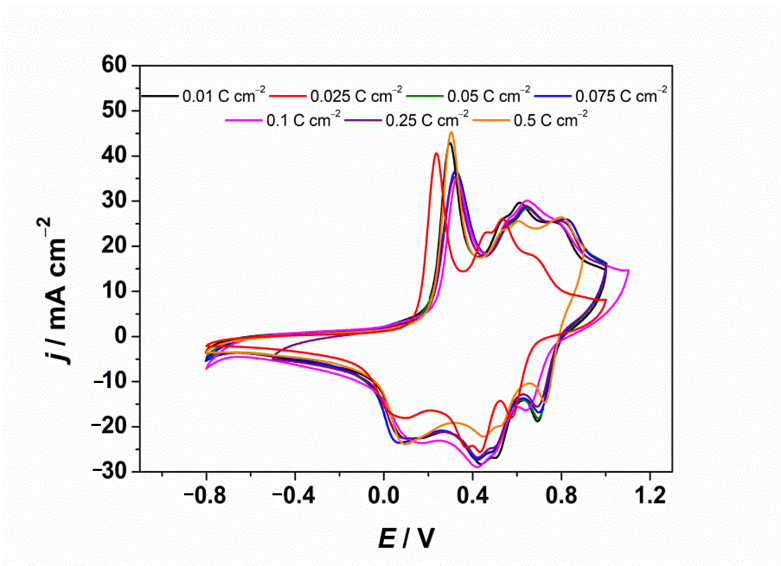


**Figure S3.** Cyclic voltammetry curves (*v* = 50 mV·s^−1^) recorded for GF/PANI/PEDOT:PSS electrode materials in 1 M H_2_SO_4_ with different charges used for electrodeposition of poly(3,4-ethylenedioxythiophene) (charge used for electrodeposition of PANI: 1.5 C·cm^−2^).


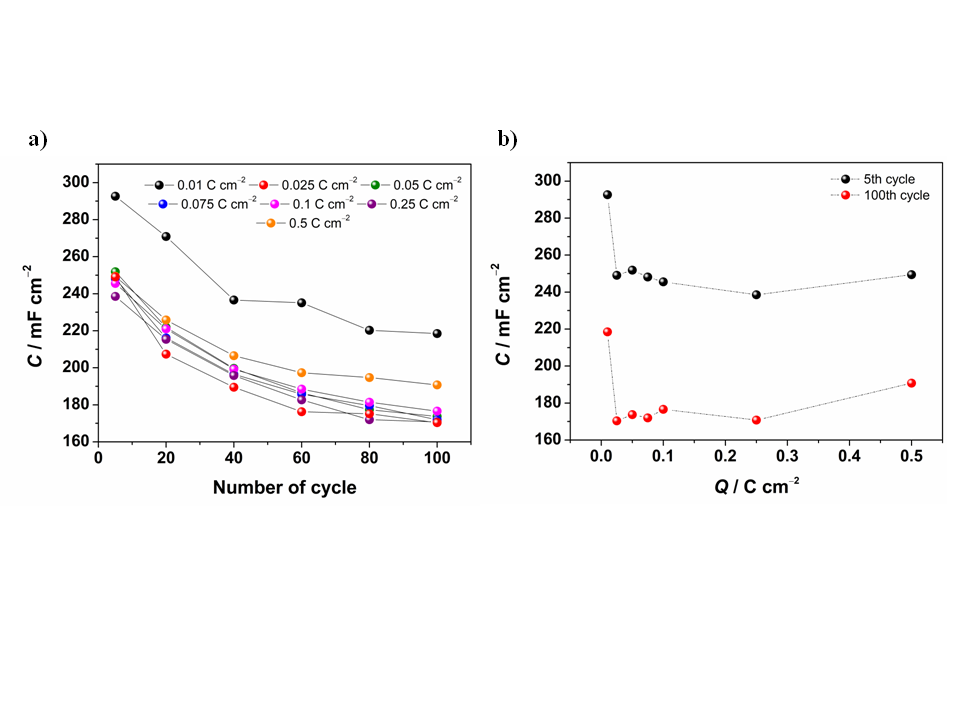


**Figure S4. (a**) Specific capacity retention after 100 cycles of GF/PANI/PEDOT:PSS electrode materials with different charges used for electrodeposition of poly(3,4-ethylenedioxythiophene) (charge at electrodeposition of PANI: 1.5 C·cm^−2^); (**b**) Specific capacity of a GF/PANI/PEDOT:PSS electrode material as a function of the charge used for electrodeposition of poly(3,4-ethylenedioxythiophene) (charge used for electrodeposition of PANI: 1.5 C·cm^−2^).


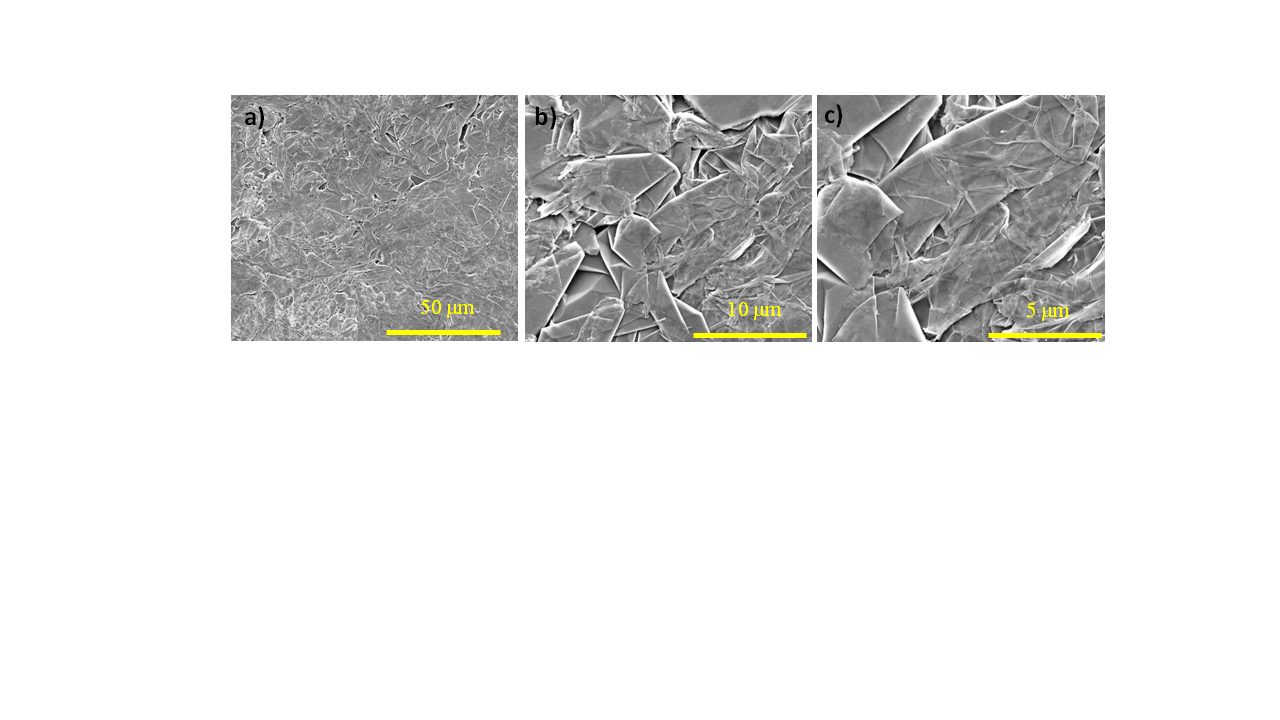


**Figure S5.** SEM image of a graphite foil with a **(a)** 1000, **(b)** 5000 and **(c)** 10,000 magnification.


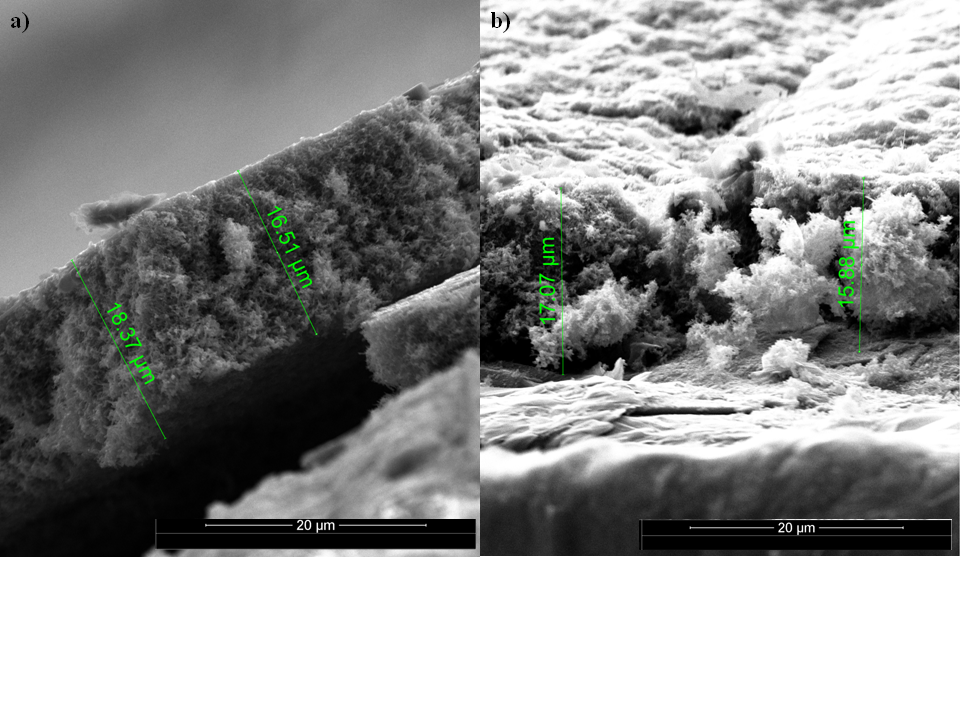


**Figure S6.** Cross section SEM images of (**a**) GF/PANI/PEDOT:PSS and (**b**) GF/PANI electrodes.


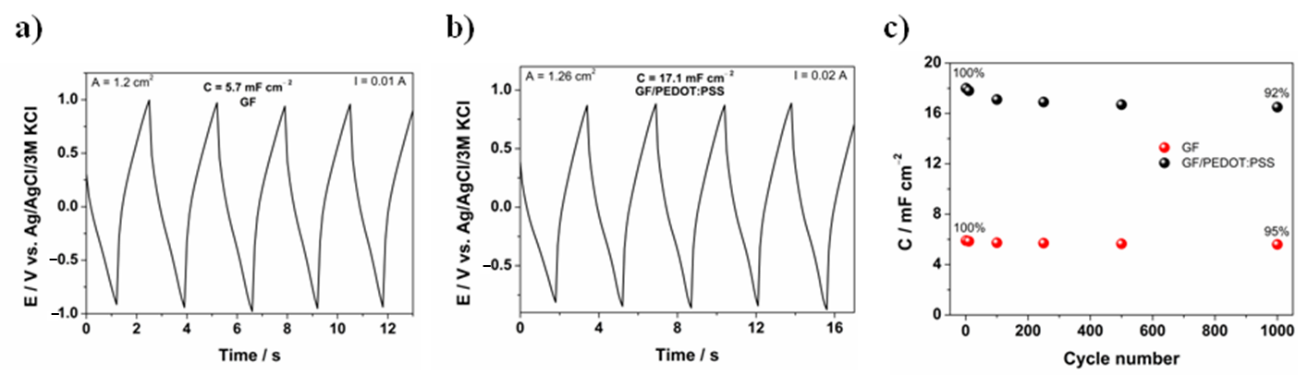


**Figure S7.** Exemplary galvanostatic charge/discharge curves of (**a**) GF and (**b**) GF/PEDOT:PSS electrodes; (**c**) Specific capacity vs. cycle number for GF and GF/PEDOT:PSS.

**Video S1.** Performance of bending procedure for a GF/PANI/PEDOT:PSS symmetric supercapacitor.

**Publisher’s Note:** MDPI stays neutral with regard to jurisdictional claims in published maps and institutional affiliations.

| 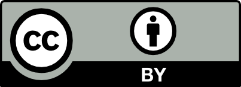 | © 2020 by the authors. Licensee MDPI, Basel, Switzerland. This article is an open access article distributed under the terms and conditions of the Creative Commons Attribution (CC BY) license (http://creativecommons.org/licenses/by/4.0/). |
| --- | --- |
